# Effectiveness of infection-containment measures on SARS-CoV-2 seroprevalence and circulation from May to July 2020, in Milan, Italy

**DOI:** 10.1371/journal.pone.0242765

**Published:** 2020-11-20

**Authors:** Valeria Cento, Claudia Alteri, Marco Merli, Federica Di Ruscio, Livia Tartaglione, Roberto Rossotti, Giovanna Travi, Marta Vecchi, Alessandro Raimondi, Alice Nava, Luna Colagrossi, Roberto Fumagalli, Nicola Ughi, Oscar Massimiliano Epis, Diana Fanti, Andrea Beretta, Filippo Galbiati, Francesco Scaglione, Chiara Vismara, Massimo Puoti, Daniela Campisi, Carlo Federico Perno

**Affiliations:** 1 Chemical-Clinical and Microbiological Analyses, ASST Grande Ospedale Metropolitano Niguarda, Milan, Italy; 2 Department of Oncology and Hemato-Oncology, Università degli Studi di Milano, Milan, Italy; 3 Infectious Diseases, ASST Grande Ospedale Metropolitano Niguarda, Milan, Italy; 4 Residency in Microbiology and Virology, Università degli Studi di Milano, Milan, Italy; 5 Department of Anesthesiology, Critical Care and Pain Medicine, ASST Grande Ospedale Metropolitano Niguarda, Milan, Italy; 6 Rheumatology Unit, ASST Grande Ospedale Metropolitano Niguarda, Milan, Italy; 7 Emergency Medicine, ASST Grande Ospedale Metropolitano Niguarda, Milan, Italy; University of Hong Kong, HONG KONG

## Abstract

**Objective:**

Through a hospital-based SARS-CoV-2 molecular and serological screening, we evaluated the effectiveness of two months of lockdown and two of surveillance, in Milan, Lombardy, the first to be overwhelmed by COVID-19 pandemics during March-April 2020.

**Methods:**

All subjects presenting at the major hospital of Milan from May-11 to July-5, 2020, underwent a serological screening by chemiluminescent assays. Those admitted were further tested by RT-PCR.

**Results:**

The cumulative anti-N IgG seroprevalence in the 2753 subjects analyzed was of 5.1% (95%CI = 4.3%-6.0%), with a peak of 8.4% (6.1%-11.4%) 60–63 days since the peak of diagnoses (March-20). 31/106 (29.2%) anti-N reactive subjects had anti-S1/S2 titers >80 AU/mL. Being tested from May-18 to June-5, or residing in the provinces with higher SARS-CoV-2 circulation, were positively and independently associated with anti-N IgG reactivity (OR [95%CI]: 2.179[1.455–3.264] and 3.127[1.18–8.29], respectively). In the 18 RT-PCR positive, symptomatic subjects, anti-N seroprevalence was 33.3% (95% CI: 14.8%-56.3%).

**Conclusion:**

SARS-CoV-2 seroprevalence in Milan is low, and in a downward trend after only 60–63 days since the peak of diagnoses. Italian confinement measures were effective, but the risk of contagion remains concrete. In hospital-settings, the performance of molecular and serological screenings upon admission remains highly advisable.

## Introduction

On January 30 2020, the World Health Organization (WHO) classified the ongoing outbreak by the Severe Acute Respiratory Syndrome Coronavirus 2 (SARS-CoV-2) in Wuhan province, China, as a Public Health Emergency of International Concern [1]. In Italy, on March 11, when WHO declared a global pandemic, the Coronavirus Disease 2019 (COVID-19) had already caused 12,462 overt infections, and 827 deaths, 58.4% of which in Lombardy region [2]. Lombardy was the first region in Europe to be affected by an important contagions’ peak during the month of March, and, still today, it suffers from the highest proportion of population infected (attack rate) among all Italian regions [2].

On March 9^th^, while the rate of diagnoses was steeply rising, the Italian government decided to shut down all unnecessary activities, and to apply a strict shelter-in-place order. After 2 months of lock-down, a “2^nd^ phase” started on May 4, with partial reopening of commercial activities, and less stringent restrictions to mobility. During the same week, the major COVID-19 reference hospital of Milan (Lombardy) started to apply a universal serological and molecular screening for SARS-CoV-2 to all subjects presenting to its Emergency Room (ER), or admitted for any reason.

For the epidemiological purpose of case-finding, territory-based mass screening is the optimum at which we all refer [3–5]. Yet, it presents such challenges to make it unlikely to be readily feasible [6]. The information provided by a systematic hospital-based screening, on the other hand, can rapidly contribute to fulfill a critical knowledge gap on SARS-CoV-2 circulation in the general population, and monitor the dynamic of local epidemics, by identifying and tracking unknown or unrecognized contact with SARS-CoV-2. Whether combined with molecular diagnostics, hospital-based screenings also constitute an essential element for the correct internal allocation of patients, ensuring high safety-standards in the management of daily clinical activities.

As of July 5, 2020, after 68,274 recognized cases, 16,697 deaths, several weeks of activities’ lock-down, shelter-in-place measures, and severe limitation of person-to-person contacts, the rate of contagion in Lombardy seems to have reached its lowest [2]. While our screening program is still ongoing, after 8 weeks since its beginning, and 4 months since the epidemic peak, we are at a sufficient time distance to effectively evaluate the population's response to viral spread, and the effectiveness of this hospital-based approach to case-finding. With such premises, we are reporting here a snapshot of SARS-CoV-2 seroprevalence, and contextual nasopharyngeal viral shedding, on 2753 consecutive patients with no known history of COVID-19, who presented themselves, or were admitted to Niguarda hospital from May 11 to July 5, 2020.

## Material and methods

### Study design and target population

From May 11, 2020, to July 5, 2020, all consecutive subjects presenting at the ASST Grande Ospedale Metropolitano Niguarda (Niguarda hospital) ER, or admitted for any reason (including day-hospitals), underwent a serologic SARS-CoV-2 testing for antibodies (Ab) targeting the nucleocapsid (N) protein of SARS-CoV-2. Subjects who were symptomatic, or in process to be admitted to Niguarda hospital for any other reason were further tested for viral-RNA shedding by RT-PCR on nasopharyngeal swabs.

Serological reactivity for Ab targeting the S1 and S2 domains of the Spike protein of SARS-CoV-2 was retrospectively analysed on residual serum samples in a subgroup of anti-N reactive subjects.

Patients who, during the period under analysis, entered our centre in the context of a post-COVID molecular follow-up, were excluded from the analysis. All subjects with symptomatic COVID-19 previously diagnosed by RT-PCR, and who were already followed or hospitalized before the start of screening, were also excluded.

The planning conduct and reporting was in line with the Declaration of Helsinki, as revised in 2013.

This study was approved by the local Research Ethics Committee (Comitato Etico Milano Area 3; prot. 92–15032020), and written informed consent was obtained.

### Laboratory methods

A chemiluminescent microparticle immunoassay intended for the qualitative detection of anti-N IgG was used for seroprevalence assessment (Abbott ARCHITECT SARS-CoV-2 IgG assay, Abbott, Abbott Park, Illinois, US). According to manufacturer’s instructions, a signal/cut-off (S/Co) ratio ≥1.4 was interpreted as reactive [7]. The specificity range of this test is 99.6%-100%; sensitivity depends on timing of testing, varying from 8.6–53.1% in the first 6 days since symptoms onset, to 43.6–82.4% between day-7 and day-14, and 84.2–100% after day-14 [7–9].

The SARS-CoV-2 S1/S2 IgG reactivity, expressed as arbitrary units (AU/mL), was evaluated by the LIAISON® SARS-CoV-2 S1/S2 IgG assay, a standardized automated chemiluminescent assay which run on a LIAISON® XL Analyzer (DiaSorin S.p.A., Saluggia, Italy). According to manufacturer’s instructions, a result ≤12.0 AU/mL was interpreted as negative, a result in the range 12.1–15 AU/mL as equivocal, and a result ≥15.0 AU/mL as positive [10]. The test’s sensitivity is time-dependent, being 25% in the first 5 days after RT-PCR-confirmed diagnosis, 90.4% from day 5 to day 15, and 97.4% from day-15 onwards [10].

SARS-CoV-2 RNA detection on nasopharyngeal swabs collected at the time of anti-N serology assessment, was performed using one of the following assays, by random choice: GeneFinder® COVID-19 Plus RealAmp Kit (OSANG Healthcare, Anyangcheondong-ro, Dongan-gu, Anyang-si, Gyeonggi-do, Korea) on ELITech InGenius® instrument (ELITech Group, Torino, Italy); AllplexTM 2019-nCoV Assay (Seegene Inc, Seoul, Korea) on Nimbus instrument (Hamilton, Agrate Brianza, Italy); XPERT® Xpress SARS-CoV-2 (Cepheid, Sunnyvale, USA) on GeneXpert Instrument System (Cepheid, Sunnyvale, USA).

### Statistical analysis

Descriptive statistics are expressed as median values and interquartile range (IQR) for continuous data and number (percentage) for categorical data, unless otherwise stated.

The overall and per week seroprevalence and 95% confidence interval (CI) were calculated by the asymptotic (Wald) method. We tested the association between variables with the Fisher exact test (for categorical variables) or Kruskal Wallis test (for continuous variables). Odds ratios (OR) and 95% CI were calculated with univariable logistic regression to assess characteristics associated with seroprevalence. Variables resulting significant in univariable were further analysed in multivariable logistic regression to assess their independent association. Missing data were excluded.

Statistical analyses were performed with SPSS software package for Windows (version 23.0, SPSS Inc., Chicago, IL). A 2-sided p-value <0.05 was considered statistically significant.

## Results

### Anti-N IgG seroprevalence at hospital presentation or admission

A total of 2824 subjects admitted to Niguarda's emergency room, or hospitalized between May 11 and July 5, 2020, were screened for SARS-CoV-2 anti-N IgG. Of them, 71 were excluded from the seroprevalence analysis for a previous known history of a laboratory-confirmed COVID-19.

The final study population thus consisted of 2753 patients.

Overall, 1389 of 2753 patients (50.1%) were male; 8 (0.3%) were younger than 10 years, 132 (4.8%) were aged 11–24 years, 786 (28.6%) were aged 25–49 years, 545 (19.8%) were aged 50–64 years, 984 (35.7%) were ages 65–84 years, and 316 (11.5%) were older than 85 years. The 92.1% of patients (n/N = 2028/2203 with available information) resided in the metropolitan area of Milan, while the 2.0% (n/N = 44/2203) resided in the provinces of Bergamo (n = 21), Brescia (n = 9), Lodi (n = 8), and Cremona (n = 6), the most affected by the SARS-CoV-2 pandemic (% infected/population >1.5% at the end of May 2020). The 87.4% of screenings (n/N = 2406/2753) were performed at admittance to the ER.

The cumulative anti-N IgG seroprevalence in the population analyzed was of 5.1% (95% CI: 4.3%-5.9%; n/N = 140/2753). Table 1 provides an overview of the weekly seroprevalence.

**Table 1:** Overview of anti-N IgG seroprevalence and SARS-CoV-2 RT-PCR positivity by screening week.

	SARS-CoV-2 anti-N IgG screening result, N		Seroprevalence and 95% confidence interval^a^	SARS-CoV-2 RT-PCR result, N		RT-PCR positivity and 95% confidence interval^a^
	Positive	Negative		Positive	Negative	
**Overall**	140	2613	5.1 (4.3–6.0)	45	2190	2.0 (1.5–2.7)
Week 1	20	526	3.7 (2.4–5.6)	4	235	1.7 (0.5–3.8)
Week 2	36	392	8.4 (6.1–11.4)	13	374	3.4 (1.9–5.5)
Week 3	22	309	6.6 (4.4–9.9)	5	304	1.6 (0.6–3.4)
Week 4	22	322	6.4 (4.3–9.5)	6	277	2.1 (0.8–4.2)
Week 5	8	295	2.6 (1.3–5.1)	1	293	0.3 (0.0–1.5)
Week 6	17	279	5.7 (3.6–9.0)	6	271	2.2 (0.9–4.3)
Week 7	6	245	2.4 (1.0–5.1)	3	228	1.3 (0.3–3.3)
Week 8	9	245	3.5 (1.9–6.6)	7	208	3.3 (1.4–6.2)

Anti-N = antibodies against viral nucleocapsid; N = number; RT-PCR = real-time polymerase chain reaction; SARS-CoV-2 = severe acute respiratory syndrome coronavirus 2.

^a^ Asymptotic (Wald) method.

**Fig 1:**
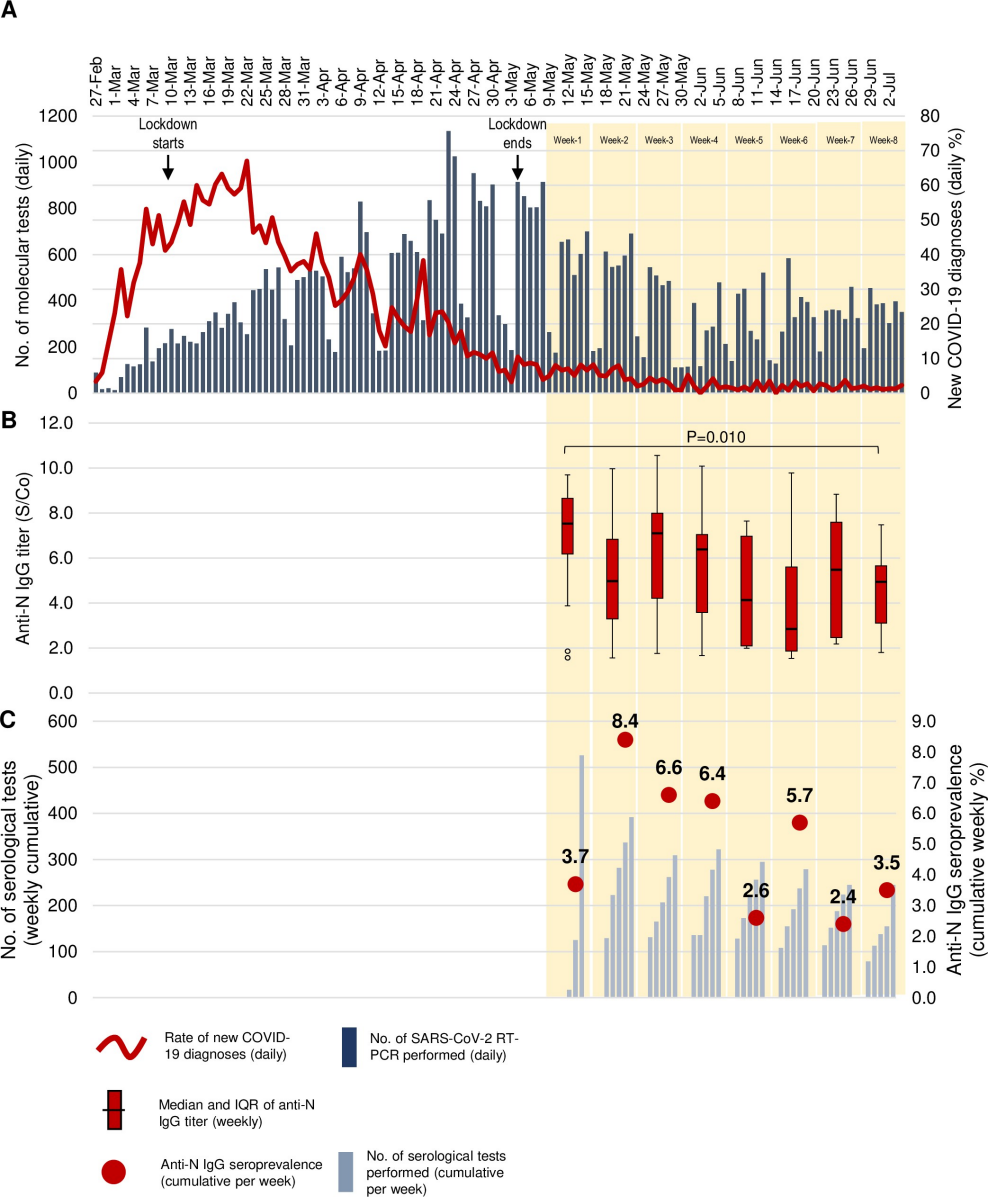
Daily rate of laboratory-confirmed COVID-19 cases (A), weekly distribution of anti-N IgG titers (B), and weekly anti-N IgG seroprevalence (C). The daily rate of first-time positive real-time PCR results on nasopharyngeal swabs are reported for each day (red line), calculated on the total number of swabs processed at ASST GOM Niguarda (dark-blue bars), from February 27, 2020, to June 26, 2020. The median and interquartile range (IQR) of anti-N IgG titers (red boxes) are calculated for each week of screening. The anti-N IgG seroprevalence (red dots) is calculated on the cumulative number of samples analyzed each week, from May 11, to June 26, 2020 (light-blue bars). Grey shading indicates the period of shelter-in-place orders (from March 9 to May 4, 2020). Orange shading shows the sampling periods for screening. P-value is calculated by Kruskal Wallis test. Anti-N = antibodies against viral nucleocapsid; COVID-19 = Coronavirus Disease 2019; No. = Number; RT-PCR = real-time polymerase chain reaction; S/Co = Sample/Cut-Off; SARS-CoV-2 = Severe Acute Respiratory Syndrome Coronavirus 2.

The first screenings were performed on May 11, at an average distance of 50 days since the peak of SARS-CoV-2 diagnoses, that we registered around March 20, 2020 (Fig 1, panel A). The distribution of the median anti-N IgG titers showed a downward trend over the 8 weeks of screening (p = 0.010; Fig 1, Panel B), in parallel with a decrease of the weekly seroprevalence (Fig 1, panel C). Indeed, during week-7 and week-8, the last two analyzed in our program, the seroprevalence decreased to 2.4% (95% CI: 1.0%-5.1%) and 3.5% (95% CI: 1.9%-6.6%), while it was 8.4% (95% CI: 6.1%-11.4%), 6.6% (95% CI: 4.4%-9.9%) and 6.4% (95% CI: 4.3%-9.5%) during week-2, week-3 and week-4, respectively (p = 0.001 by Chi^2^ test for trend). The shorter time distance since the peak of contagions can contribute for the lower seroprevalence observed during week-1 (3.7%, 95% CI: 2.4%-5.6%).

The univariate and multivariate logistic regression models showed that being tested from May 18 to June 5, 2020, as well as residing in the provinces of Brescia, Bergamo or Cremona, were positively and independently associated with an increased chance to be anti-N IgG reactive (OR 2.179 [95% CI: 1.455–3.264] and 3.127 [95% CI: 1.18–8.29], respectively; Table 2). The rate of anti-N IgG reactivity was instead similar between men and women (OR 1.259 [95% CI: 0.895–1.772], p = 0.186), and for different age groups (Table 2).

**Table 2:** Potential predictors of anti-N IgG seropositivity. Model adjusted for age, sex, province of residence, time of screening, and ward of admittance.

	SARS-CoV-2 anti-N IgG screening result		Odds ratio (95% CI)	P-value
	Positive	Negative		
**Age groups (years), n (%)**				
≤10 (N = 8)	0 (0%)	8 (100%)	-	
11–24 (N = 132)	11 (8.3%)	121 (91.7%)	1.989 (0.999–3.961)	0.050
25–49 (N = 786)	50 (6.4%)	736 (93.6%)	1.487 (0.978–2.260)	0.064
50–64 (N = 545)	24 (4.6%)	503 (95.4%)	1.044 (0.626–1.741)	0.868
65–84 (N = 984)	43 (4.4%)	941 (95.6%)	1 (ref)	
≥85 (N = 316)	12 (3.8%)	304 (96.2%)	0.864 (0.45–1.659)	0.660
**Sex, n (%)**				
Female (N = 1364)	77 (5.6%)	1287 (94.4%)	1.259 (0.895–1.772)	0.186
Male (N = 1389)	63 (4.5%)	1326 (95.5%)	1 (ref)	
**Province of residence, n (%)**^**a**^				
Brescia, Bergamo or Cremona (N = 36)	5 (13.9%)	31 (86.1%)	3.127 (1.18–8.29)	0.022
Milan (N = 2028)	97 (4.8%)	1931 (95.2%)	1 (ref)	
**Date of SARS-CoV-2 screening, n (%)**				
Before May 18 (N = 546)	20 (3.7%)	526 (96.3%)	1 (ref)	
Between May 18 and June 5, 2020 (N = 1103)	80 (7.3%)	1023 (92.7%)	2.138 (1.195–3.825)	0.01
After June 5, 2020 (N = 1104)	40 (3.6%)	1064 (96.4%)	0.954 (0.503–1.810)	0.885
**SARS-CoV-2 screening performed in the ER, n (%)**				
Yes (N = 2406)	122 (5.1%)	2284 (94.9%)	0.976 (0.587–1.623)	0.926
No (N = 347)	18 (5.2%)	329 (94.8%)	1 (ref)	

Data are n (%) unless otherwise stated. Odds ratio (95% CI) for anti-N IgG reactivity by univariate logistic regression analysis are shown for age groups, sex and ER screening; odds ratio (95% CI) for anti-N IgG reactivity by multivariate logistic regression analysis are shown for province of residence and date of SARS-CoV-2 screening. Age 65–84 years, male, residence in Milan, screening date Before May 18, and screening performed in hospital wards other than ER, are the reference groups, with which other groups are compared.

^a^Only patients with known residence in Lombardy provinces (N = 2203) are included in this analysis.

Anti-N = antibodies against viral nucleocapsid; CI = Confidence interval; ER = Emergency Room; N = number; SARS-CoV-2 = severe acute respiratory syndrome coronavirus 2.

A further analysis of the antibody response against SARS-CoV-2, performed by evaluating the anti-S1/S2 IgG reactivity on 106/140 anti-N reactive subjects, showed that 29.2% (n/N = 31/106) had contextually developed anti-S1/S2 titers above 80.1 AU/mL, 30.2% (n/N = 32/106) had titers between 40.1 and 80 AU/mL, and 27.4% (n/N = 29/106) had titers between 15.1 and 40 AU/mL. The 13.2% (14/106) was non-reactive. The cross-sectional analysis of anti-S titers did not show a significant increase or decrease in value in relation to the screening week (p = 0.209, S1 Fig).

### Molecular SARS-CoV-2 screening by real-time PCR at hospital admission

All 2235 subjects who were admitted to hospital care, regardless of their reactivity at Ab-screening, were further tested for nasopharyngeal SARS-CoV-2 shedding by RT-PCR, and 45 (2.0%) resulted positive (Table 1). Of them, 18 (40.0%) had typical COVID-19 symptoms at the time of hospital admission and serological screening, 6 (13.3%) were currently asymptomatic but retrospectively described the occurrence of symptoms compatible with COVID-19, while the last 21 (46.7%) reported a completely silent history for any COVID-related symptomatology.

The weekly rate of positive RT-PCR results remained low, yet stable, through the whole screening duration (Table 1).

In RT-PCR negative subjects, anti-N IgG seroprevalence was 5.1% (95% CI: 4.2%-6.1%) vs. 62.2% (95% CI: 47.4%-75.4%) in RT-PCR positive patients.

Anti-N IgG seroprevalence was 33.3% (95% CI: 14.8%-56.3%) in RT-PCR positive symptomatic patients, but increased to 66.7% (95% CI: 28.1%-93.5%) in clinically recovered RT-PCR positive subjects, and to 85.7% (95% CI: 67.0%-96.2%) in RT-PCR positive patients with no clinical history of a respiratory syndrome, in whom the timing of viral infection could not be precisely defined (p<0.001).

None of the 22 RT-PCR positive and anti-N IgG reactive asymptomatic subjects developed a respiratory syndrome while hospitalized. Furthermore, as of July 5, the frequency of confirmed RT-PCR negativization (2 consecutive negative results) at 14-day since first positivity was significantly higher in anti-N IgG positive patients (72.2% n/N = 13/18 with available molecular follow-up), than in anti-N IgG negative patients (30.0%, n/N = 3/10; p = 0.05).

## Discussion

As of July 2020, Lombardy is currently in an optimal time condition for a seroprevalence analysis, and to check whether the containment measures applied have in any way allowed to limit viral spread.

Our region was the first, in Europe, to be intensively affected by SARS-CoV-2 infection. Recent phylogenetic estimates suggest that the introduction of SARS-CoV-2 in Lombardy occurred, with multiple hits, since the end of January 2020 [11], a hypothesis supported by the finding of a 2% serological positivity in blood donors who donated before February 20, 2020, in the Lodi area [12]. In our hospital, the largest of Milan and the second largest in Lombardy, the daily rates of new COVID-19 diagnoses reached their maximum around March 20, 2020, compatibly with a peak of contagions during the 1^st^-2^nd^ week of the same month. After 2 months of complete lock-down and shelter-in-place orders (from March 9 to May 4, 2020), and 8 weeks of serological monitoring after a cautious reopening (from May 11 to July 5, 2020), our snapshot analysis of SARS-CoV-2 seroprevalence revealed that the 5.1% (95% CI = 4.3%-5.9%) of 2753 subjects tested have entered into contact with the virus. This estimation is in line with the result of a Spanish nationwide screening program, based on the same test we used, that reported an overall anti-N IgG seroprevalence of 4.6% [4]. Yet, it exceeds of at least 5-fold the number of laboratory-confirmed COVID-19 diagnoses in Lombardy and Milan, that, up to July 4, corresponded to the ~1% of the overall population.

Our data also indicate that we are on an epidemic tail that is not yet completely exhausted. During the 8 weeks of screening, the 2.0% of the subjects tested were RT-PCR positive, and 40.0% of them had symptomatic COVID-19. Moreover, even though most of the subjects who developed anti-N IgG were also anti-S1/S2 IgG reactive, the titers of this latter antibody class exceeded the 80 AU/mL only in about 30% of cases. This suggests that even those who developed an antibody response against the virus, would not necessarily be protected against a second wave of contagions that could emerge from the residual diffusion we are witnessing.

Overall, our findings are in agreement with previous studies in demonstrating that the vast majority of the population is still immunologically naïve [4, 13, 14], and contribute to support the concept of a lack of herd immunity (as somewhat expected). At the same time, our low seroprevalence estimate also suggests that the containment measures implemented have been useful in limiting the spread of the infection in Milan. In our analysis, subjects residing in the Lombardy’s provinces that suffered from a higher epidemic peak, such as Brescia, Bergamo or Cremona, had a higher risk of being anti-N reactive, compared to those residing in Milan. This is in agreement with very recent phylogenetic evidences of an effective containment of viral spread within specific geographical areas, with little mixing between different provinces in Lombardy [11].

Another evidence in support to the effectiveness of the containment measures adopted, comes from the distribution of the seroprevalence rates during the 8 weeks of screening. Being screened between May 18 and June 5 (2–3 months after the peak of diagnoses) was associated with the higher chance of being anti-N reactive. During this time-frame, the weekly seroprevalence ranged between 8.4% and 6.6%. afterwards, it \progressively and significantly decreased after June 5, down to a 3.5% in the 245 subjects screened from June 29 to July 3, 2020. This distribution seems to follow what has been the shape and duration of our COVID-19 epidemic curve, drastically cut down following the introduction of stringent contain measures.

By reasonably assuming that the seroprevalence rate we observed during our 8 weeks of screening are largely referring to infection events that occurred before the lockdown, the decreasing trend of the absolute value of anti-N antibody titers measured over time also leads us to believe that these can have a fairly rapid decay kinetics. A kinetics that, to date, does not seem to be as rapid for anti-S1/S2 IgG, whose titers did not show a similar decay in the time-frame we analyzed. This hypothesis is supported by the results of a recent prospective study, that estimated a mean half-life of anti-N IgG responses following SARS-CoV-2 infection of only 52 (95% CI 42–65) days vs. a half-life of 81 (95% CI 61–111) and 83 days (95% CI 55–137) for the S- and RBD-antibody responses, respectively [15].

One reason for this finding could lie in the delay of the anti-S IgG peak, which represents an antibody class developed in more advanced stage of coronaviruses infection than the anti-N [16, 17]. The time window we analyzed may therefore not be large enough to appreciate the fall of the anti-S titer.

Yet, eventually, a decline in serum titer is expected for both types of antibodies. In 2006, Liu et al. demonstrated that SARS-CoV-1 IgG and neutralizing antibodies peak after 4 months since infection, and then progressively decreased over time [18]. Similarly, Long QX et al., observed that SARS-CoV-2 IgG levels and neutralizing antibodies start to decrease within 2–3 months after infection [19], supporting estimated IgG half-lives lower than 90 days [15].

These emerging data thus stress the concept that, to reliably intercept the exposed population, serological screening needs be carefully centered on an appropriate time distance since the epidemic peak, as the search for certain type of SARS-CoV-2 antibodies for epidemiological purpose may have a range of usefulness limited in time [15].

The definition of the correct screening timing, and methods, is thus of fundamental importance, especially in hospital settings, where failure to control possible sources of infection, and to consequently prevent viral spread, can have dramatic consequences. Although the search for anti-N Ab could be advantageous in terms of initial sensitivity [20, 21], the poor ability to identify antibody production in the early stages of SARS-CoV-2 infection is a drawback shared by all the serological tests available today [22], including the one we used. In our study, anti-N IgG seroprevalence was only 33.3% in RT-PCR positive symptomatic subjects, and increased to 66.7% in those clinically-recovered. Even though we could not estimate the duration of symptoms before the performance of the test, nor assess the actual infectivity of currently asymptomatic subjects, this observation supports a lower rate of anti-N reactivity in more recent infections [7–9], an aspect that should be acknowledged while using serological screening as the only tool to evaluate a contact with SARS-CoV-2 at hospital admittance. The use of a lower cut-off (1.0 or 0.8 S/Co ratio) for defining anti-N reactivity with the Abbott Architect assay has been recently proposed as a strategy to increase sensitivity [8], even though at possible expenses of specificity [23]. In our study, such cut-off reduction would have led to a slight increase of the anti-N reactivity rate in patients with positive RT-PCR, from 62.2% to 68.9%. This limited improvement, and the small number of patients analysed, prevent us from stating any conclusion on this working hypothesis. As long as there is no further supporting data, the use of the positivity cut-off recommended by the manufacturer seems to be a reasonable strategy.

Although it was not the primary objective of the study, our data confirm what is known for other respiratory infections: serology is positive in those who have had the infection (more or less symptomatic), but it does not allow an early diagnosis in those who are presenting the first symptoms nor—probably–allows the identification of those at greater risk of contagion. It being understood that RT-PCR remains the diagnostic gold-standard [24, 25], serology can still provide useful elements, in clinical practice, to evaluate the prospect of COVID-19 evolution, and to optimize the intra-hospital allocation of first-time-positive asymptomatic patients. In our clinical practice, the 72.2% of Ab-positive/PCR-positive patients experienced RT-PCR negativization within 2 weeks since diagnosis (vs. 30.0% of Ab-negative/PCR-positive), and none of the 22 asymptomatic patients ever developed a respiratory syndrome during subsequent hospitalization. When hospitalizing a PCR-positive subject for reasons other than COVID-like symptoms, a positive serology at diagnosis thus reasonably supports the ongoing resolution of SARS-CoV-2 infection, with a consequently low likelihood of viral transmission, as testified by a generally rapid negativization of RT-PCR. On the other hand, a negative serology may imply a risk for a persistent RT-PCR positivity and contagiousness, and it does not exclude the possibility of a subsequent development of a respiratory syndrome, which must be taken into consideration while deciding hospitalization and monitoring protocols.

Our study has its limitations, most of which related to the known pitfalls in terms of sensitivity of currently available serological assays, and gap of knowledge on the longevity of the SARS-CoV-2 antibody response [22, 26]. The sensitivity of the Abbott’s assay we used for anti-N detection was recently shown to declined with time, to ~70% at >81 days [21]. Whether confirmed by further studies, this technical issue should be taken into account for the interpretation of the Ab decay kinetics, even though the short time-range of currently available studies (including our) has probably greatly limited its potential influence on seroprevalence estimations. Similarly, even though waning of antibody level is well described, this may not fully account for the decrease in seroprevalence. Even admitting the effective limitation of the viral spread in terms of time and space, other contingent factors may have contributed to this phenomenon, including, first of all, the causal variability of the population pertaining to our hospital. This is a study based on a single center which, although representing the largest COVID-19 reference center in Milan, is part of an area less affected by the pandemic than other Lombard provinces. The seroprevalence that we obtained, therefore, is primarily representative of the population of Milan. In addition, the population we screened is enriched with subjects with comorbidities, as referring to a hospital environment. These accounted for at least of 12.6% of our overall. Even though being screened during elective admission in chronic diseases vs. been screened in the ER was not significantly associated with a higher Ab positivity rate, we could not exclude that they had a greatest risk of contracting COVID-19 (given the frequent and continuous access to medical care), and thus that our estimated seroprevalence may not be fully superimposable on that of the general population. Lastly, even though the specificity of the serological test we used ranges between 99.6%-100% [7–9], we cannot exclude the occurrence of false-positive results that may have falsely increased our seroprevalence estimation, especially in the setting of the low seroprevalence we witnessed.

In conclusion, this hospital-wide SARS-CoV-2 screening performed by the largest hospital in Milan revealed a SARS-CoV-2 anti-N seroprevalence of 5.1% (95% CI = 4.3%-5.9%), and a detection of anti-S titers >80 AU/mL in no more that 30% of anti-S positive subjects. In line with the reduction of the number of COVID-19 related hospital admissions in the last two months, our findings suggests that confinement measures in Lombardy were effective. Yet, we cannot count on the reduction of susceptible individuals to play a major role in slowing transmission in the months to come. The low (but not negligible) incidence of new COVID-19 diagnoses after the end of the shelter-in-place restrictions indicates that the attention to the risk of contagion must remain high. To this end, great care should be taken in selecting the most appropriate diagnostic assay, as each of them suffers from limitations that should be acknowledged when interpreting results. Upon hospital admission, serological screening alone may not be sensitive enough to ascertain a potentially infectivity. In terms of public health, it appears we therefore have no alternatives to RT-PCR for diagnosis and, above all, isolation of cases.

## Supporting information

S1 Fig Weekly distribution of anti-S1/S2 IgG titers. The median and interquartile range (IQR) of anti-S1/S2 IgG titers (grey boxes) are calculated for each week of screening. Anti-S1/S2 = antibodies against S1 and S2 domains of the Spike protein of SARS-CoV-2. AU/mL, arbitrary units per millilitre.
(TIF)
