# Supplementary figures and images for: Effectiveness of infection-containment measures on SARS-CoV-2 seroprevalence and circulation from May to July 2020, in Milan, Italy
Source: PLoS One. 2020 Nov 20;15(11):e0242765. doi: 10.1371/journal.pone.0242765 (PMC7679019; doi:10.1371/journal.pone.0242765)

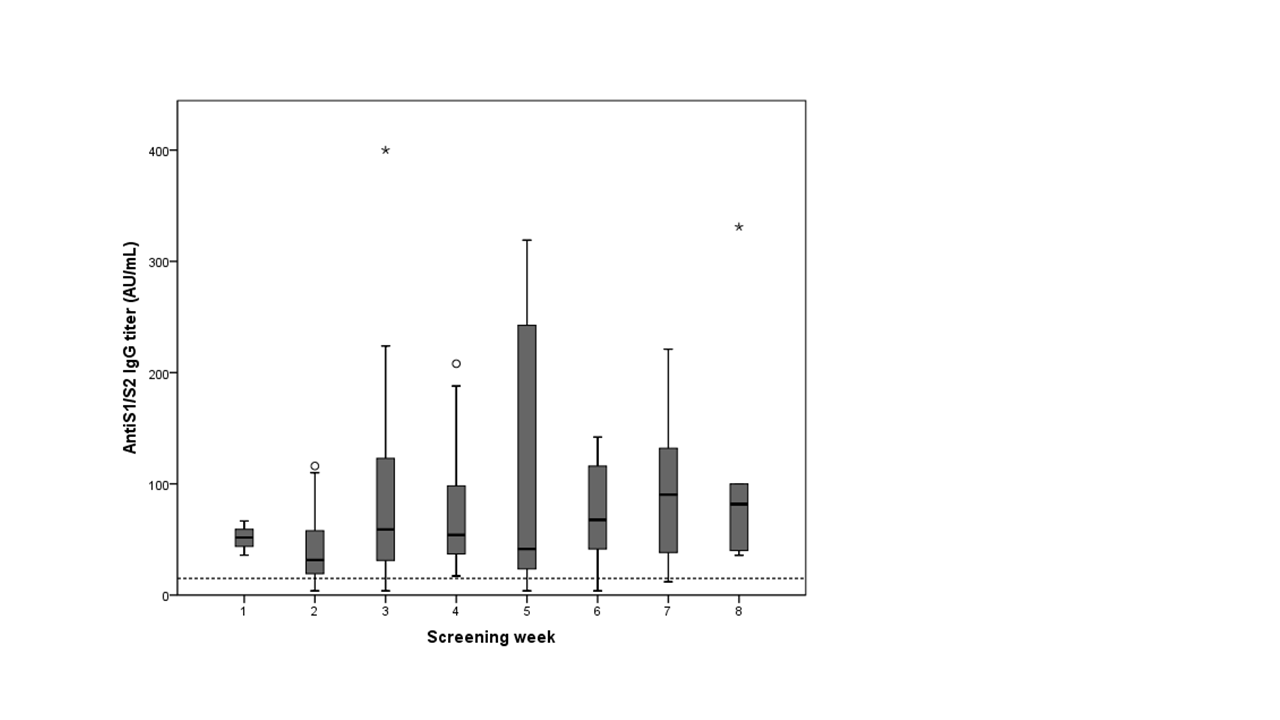

Supplement: S1 Fig — The median and interquartile range (IQR) of anti-S1/S2 IgG titers (grey boxes) are calculated for each week of screening. Anti-S1/S2 = antibodies against S1 and S2 domains of the Spike protein of SARS-CoV-2. AU/mL, arbitrary units per millilitre. (TIF) [file pone.0242765.s001.tif]
